# Comprehensive Gene Expression Analysis of Human Embryonic Stem Cells during Differentiation into Neural Cells

**DOI:** 10.1371/journal.pone.0022856

**Published:** 2011-07-28

**Authors:** Ali Fathi, Maryam Hatami, Vahid Hajihosseini, Faranak Fattahi, Sahar Kiani, Hossein Baharvand, Ghasem Hosseini Salekdeh

**Affiliations:** 1 Department of Molecular Systems Biology, Cell Science Research Centre, Royan Institute for Stem Cell Biology and Technology, Avicenna Research Institute (ACECR), Tehran, Iran; 2 Department of Stem Cells and Developmental Biology, Cell Science Research Centre, Royan Institute for Stem Cell Biology and Technology, Avicenna Research Institute (ACECR), Tehran, Iran; 3 Department of Biotechnology, College of Science, University of Tehran, Tehran, Iran; 4 Department of Developmental Biology, University of Science and Culture, Avicenna Research Institute (ACECR), Tehran, Iran; 5 Department of Systems Biology, Agricultural Biotechnology Research Institute of Iran, Karaj, Iran; University of Pittsburgh School of Medicine, United States of America

## Abstract

Global gene expression analysis of human embryonic stem cells (hESCs) that differentiate into neural cells would help to further define the molecular mechanisms involved in neurogenesis in humans. We performed a comprehensive transcripteome analysis of hESC differentiation at three different stages: early neural differentiation, neural ectoderm, and differentiated neurons. We identified and validated time-dependent gene expression patterns and showed that the gene expression patterns reflect early ESC differentiation. Sets of genes are induced in primary ectodermal lineages and then in differentiated neurons, constituting consecutive waves of known and novel genes. Pathway analysis revealed dynamic expression patterns of members of several signaling pathways, including NOTCH, mTOR and Toll like receptors (TLR), during neural differentiation. An interaction network analysis revealed that the TGFβ family of genes, including LEFTY1, ID1 and ID2, are possible key players in the proliferation and maintenance of neural ectoderm. Collectively, these results enhance our understanding of the molecular dynamics underlying neural commitment and differentiation.

## Introduction

Embryonic stem cells (ESCs) are a promising tool for the study of neural development and cell lineage specification. The current lack of knowledge about cues for mammalian neuronal commitment and differentiation is at least partly due to a paucity of available model systems that permit direct observation of developmental processes. Studies of the molecular mechanisms underlying the neural differentiation of human ESCs (hESCs) can help to unravel the complex gene pathways that are involved in neural cell commitment and differentiation processes. High throughput studies of gene expression have been applied to neural stem cells (NSCs) derived from the subventricular zone (SVZ) [1], NSCs derived from mouse ESCs and the fetal brain [2], dopaminergic neurons from mouse ESCs [3], heterogeneous neural cells from mouse ESCs [4] and neural progenitors (NPs) differentiated from mouse ESCs [5] and forebrain periventricular zone NPs compared to neuroectoderm from mESCs [6]. Cytoskeletal genes, cell membrane receptor genes, and transcription factor genes are differentially expressed in most cell types. Recently, Wu et al. (2010) analyzed the transcriptome of hESCs during differentiation into the neural lineage at the early initiation, neural progenitor, and early glial-like stages [7] and found an extraordinary degree of stage-specific transcription and splicing. The diversity was highest in undifferentiated hESCs and decreased upon differentiation [7].

Previously, we described the differentiation of hESCs into NPs and neural cells with typical cellular, molecular and ultrastructural markers using a defined adherent culture protocol [8], [9]. Here we analyzed the transcriptome of hESCs during differentiation into neural cells [8] using a whole genome microarray chip. We identified differentially expressed genes that may be linked to neural fate specification, proliferation, and differentiation. Our data extend the gene expression network for neural differentiation and reveal novel aspects of transcriptional control pathways underlying the multistep process of commitment and differentiation of hESCs into neural cells.

## Materials and Methods

### Human ESC culture and sample preparation

The hESC line Royan H6 [10] was passaged and cultured under feeder-free culture conditions on Matrigel in hESC medium containing DMEM/F12 medium supplemented with 20% knock-out serum replacement, 2 mM L-glutamine, 1% nonessential amino acids, 100 units/ml penicillin and 100 µg/ml streptomycin, insulin-transferrin-selenite, (All from Invitrogen), 0.1 mM β-mercaptoethanol and 100 ng/ml basic-fibroblast growth factor (bFGF, Royan Institute). The cells were grown in 5% CO2 and 95% humidity, and they were further passaged every 7 days. For passaging, hESCs were treated with collagenase IV (0.5 mg/ml, Invitrogen): Dispase (1 mg/ml, Invitrogen) at 37°C for 5–7 min then the enzyme was removed and washed with PBS. Cells were collected by gently pipetting and replated on matrigel coated dishes and the medium changed every other day [8].

### Neural differentiation

Neural differentiation was induced as described previously [8]. Briefly, seven-day hESCs were induced to neural phenotype by 20 ng/ml bFGF, retinoic acid (RA, 2 µM, Sigma-Aldrich), Noggin (500ng/ml, R&D), Shh (50 ng/ml, R&D), and leukemia inhibitory factor (LIF, 10 ng/ml, chemicon) for two days (NI stage). Cells were incubated for an additional seven days without Noggin and SHH until neuroectodermal islands with columnar cells, known as rosette structures, appeared (NE stage). For a further week RA removed from the medium and during this time, the rosette structures changed into neural tube-like structures (NT stage). These structures were separated manually from the surrounding flat cells with a sterile pulled-glass pipette under the phase contrast microscope (10X, Olympus, CKX41). The neural tube-like structures were dissociated into single cell by 0.01% trypsin (Invitrogen)/2 mM disodium EDTA and then replated on laminin (5 µg/ml, Sigma-Aldrich) and poly L-Ornithine (15 µg/ml, Sigma-Aldrich)-coated tissue culture dishes to generate differentiated neural cells (DN stage) in neurobasal medium, supplemented with 2% N2 and 2% B27, 2.5% fetal bovine serum (All from Invitrogen), ascorbic acid (AA, Sigma-Aldrich), fibronectin (5 µg/ml, Sigma-Aldrich), laminin (1 µg/ml, Sigma-Aldrich), BDNF(20 ng/ml, R&D), GDNF(20 ng/ml, Sigma-Aldrich) and db-cAMP(1 ng/ml, Sigma-Aldrich) for up to 14 days.

### Immunofluorescence staining and flow cytometry analysis

To perform immunofluorescence staining, cells were fixed in 4% paraformaldehyde for 20 minutes, permeabilized with 0.2% Triton X-100 for 10 minutes and blocked in PBS with 10% serum of host which secondary antibody was derived in PBS for 1 hour. Cells were incubated with primary antibody for 1 hour at 37°C, washed, and incubated with fluorescein isothiocyanate (FITC)-conjugated secondary antibodies for 1 hour at 37°C. The primary antibodies used were Oct4 (1∶100, Santa Cruz Biotechnology, SC-5279) and Nanog (1∶100, Santa Cruz Biotechnology, SC-30331) for undifferentiated hESC determination and anti-Nestin, Sox1, Pax6, microtubule-associated protein monoclonal IgG (MAP2), glial fibrillary acidic protein (GFAP), serotonin, and tyrosine hydroxylase (TH) for differentiated cell determination. The complete information for and concentrations of the antibodies have been described previously [8]. The nuclei were counterstained with DAPI or propidium iodide (PI). Cells were analyzed with a fluorescence microscope (Olympus, Japan). Flow cytometry analysis for Nestin, Sox1, and Pax6 was performed as described previously [8].

### Electrophysiology analysis

Whole cell patch clamp recording method was used for functional testing of differentiated cells in 30 days post induction which cultured on cover slip. The record was carried out in the room temperature (25°C) and cell currents were recorded in voltage clamp mode. Patch electrode (Filament borosilicate glass, 1.5 mm outer diameter, HARVARD apparatus, GC150F-10) resistance brought up to 3 to 5 M.Ώ and pulled used horizontal puller (Sutter Instruments P-97 USA). Recorded signal amplified and filtered (2 KHz low-pass Bessel Filter) using Multiclamp 700B amplifier (Axon instrument, USA). Amplified signals were acquired at 10 KHz using a Digidata 1440 analog-to-digital (A/D) board and PClamp 10 software (Axon instrument, USA).

All voltage protocol will describe in result section. The signals analyzed by Clampfit10 software (Axon instrument, USA) in off-line mode. For recording cell currents the bath solution was used (mM): Nacl 160, CaCl2 2, HEPES 10, D-Glucose 10. The solution pH adjusted in 7.4 by using NaOH and osmolarity kept around 300 mOsm The pipette solution contained: CsCl2 (130 mM), MgCl2 (2 mM), TEA-Cl (20 mM), EGTA (10 mM), HEPES (10 mM), and D-Glucose (10 mM) adjusted to PH 7.2 by using KOH and osmolarity kept around 300 mOsm. Nifedipin (5 mM) had been used to block L type Ca^++^ channels that indicated and administrated via super fusion using a fast-exchange perfusion system (ALA instruments).

### Illumina bead chip hybridizations and analysis of expression data

Differentiated cells that were representative of NI (day 2), NE (day 9) and DN (day 30) and control hESCs were generated in triplicate. Total RNA was isolated using Trizol reagent (Invitrogen). Approximately 400 ng of total RNA from three biological replicates per time point served as the input to generate biotin-labeled cRNA using a linear amplification kit (Ambion, Austin, TX, United States). RNA and biotinylated cRNA concentrations were confirmed with Nanodrop ND-1000 and controlled for quality using a BioRad Experion electrophoresis station. Next, cRNA samples (750 ng) were hybridized onto Illumina Sentrix® HumanHT-12 v3 Expression Bead Chips at 58 °C overnight (19 h). Chips were scanned with the Illumina Bead Array Reader (Factor = 1, PMT = 520, Filter = 100%), and the numerical results were extracted with GenomeStudio using the Gene Expression Module v.1.0.6. Raw data were background-subtracted and normalized using the quantile normalization method (lumi software package) [11]. Normalized data were filtered for genes with significant expression levels compared to negative control beads. Selection for differentially expressed genes was performed on the basis of arbitrary thresholds for fold changes plus statistical significance according to the Illumina t-test error model (limma software) [12]. The mRNA array data in MIAME compliant and has been submitted to the NCBI Gene Expression Omnibus (GEO) database (Accession: GSE28633).

### Data analysis

All significantly expressed transcripts (P<0.05, FC≥1.5) were clustered using a hierarchical clustering method. The determination of the correct number of clusters was based on measuring the similarity of each gene to its own cluster compared to the similarity of the gene to genes in other clusters, which was measured using the average of the intracluster and intercluster distances. MATLAB software (v. 7.3) was used for clustering and correlation. Expander software (v. 5.07) [13] was used for the hierarchical clustering of transcripts overexpressed in each stage separately and cell cycle associated transcripts. Briefly, the fold changes of the expression values compared to the ESC stage were imported into the software and standardized with a mean of 0 and a variance of 1. Then, using the average linkage method, transcripts were clustered, and the expression matrix was visualized with a dendrogram.

The STRING database (version 8.1) [14] was used to construct a regulatory network of differentially expressed transcripts. Then, a regulatory sub-graph was extracted from this network by selecting edges with inhibitory or activatory regulatory interactions. The visualization of networks was performed using Cytoscape (version 2.6.3) [15].

We used BiNGO (a Cytoscape plugin) [16] to find statistically over- or underrepresented Gene Onthology (GO) categories in the biological data as a tool to enrich the analysis of the transcriptome dataset. Enrichment was determined in reference to all human Entrez GeneIDs that were annotated in the Biological Process branch (14,394 genes total). P-values were derived from a hypergeometric test followed by the Benjamini and Hochberg false discovery rate [17]. A P-value cutoff of 0.01 was used to identify significantly enriched categories. Pathway analyses were assigned with the ClueGO (v. 1.2) plugin to all of the genes using the KEGG database. A two sided hypergeometric test was used as statistical test for the probability of each gene falling into a pathway.

### Real-Time PCR

Reverse transcription of the isolated RNA was carried out using the MMLV reverse transcriptase (USB) and oligo-dT primer according to the manufacturer's instructions. Real-time PCR was carried out on an Applied Biosystems 7900 instrument in 25 µl reactions containing 12.5 µl of SYBR Green PCR mix (Applied Biosystems, Foster City, CA, United States) and each primer at 0.375 µM. All primers used for the assays were tested for specificity and amplification efficiency. The sequences of the primers used are listed in Table S1. Relative mRNA levels were calculated using the comparative CT method, as described by the manufacturer (Applied Biosystems, Foster City, CA, United States), with GAPDH as an internal control for normalization.

### Western blot analysis

For each sample, 25 µg of protein was separated by 12% SDS-PAGE (120 V for 1 h) using a Mini-PROTEAN 3 electrophoresis cell (Bio-Rad), and the proteins were transferred to a PVDF membrane (Amersham) using a semi-dry blotting method (Bio-Rad) and Dunn carbonate transfer buffer (10 mM NaCHO_3_, 3 mM Na_2_CO_3_, 20% methanol). Membranes were blocked for 1.5 h using Western Blocker solution (Sigma, W0138) and then incubated overnight at 4°C with the respective primary monoclonal antibodies, mouse anti-TH (Sigma, 1∶10,000), rabbit anti-OTX2 (Abcam, 1 ug/ml) or mouse anti-Nestin (Chemicon, 1∶1,000). Membranes were then incubated with peroxidase-conjugated secondary antibodies, anti-mouse IgG (1∶5,000, Sigma, A9044) or anti-rabbit IgG (1∶10,000, Sigma, A2074), for 2 hr at room temperature. Finally, the blots were visualized using ECL detection reagents (Sigma, CPS-1-120). Subsequently, the films were scanned with a densitometer (GS-800, Bio-Rad). To insure that a uniform amount of protein was loaded onto the gels, the membranes were stained for total protein with Fast Green (FCF, Sigma-Aldrich, F7252).

## Results and Discussion

### Differentiation of hESCs into neural cells

**Figure 1:**
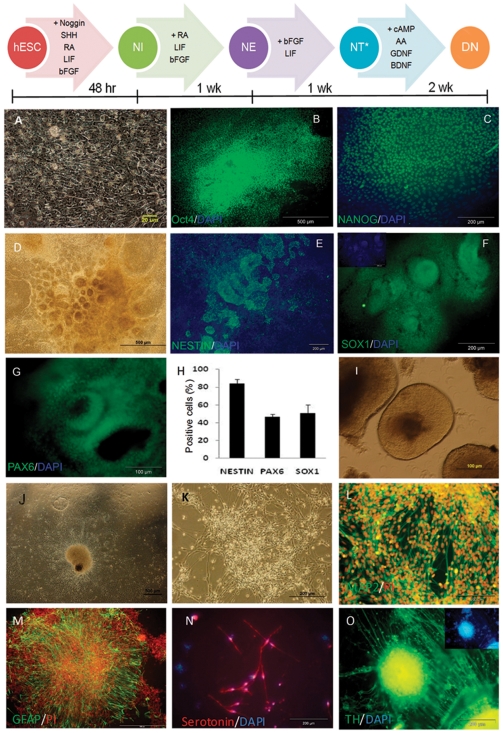
Characterization of undifferentiated and differentiated hESCs toward neural phenotype. Schematic representation of neural differentiation procedure and stages NI, NE, NT and DN in the upper panel. (A) Phase contrast photomicrographs of a colony of hESCs grown under feeder-free conditions. The cells show a high ratio of nucleus to cytoplasm and a compact morphology with clear borders. Immunofluorescence analysis of key hESC markers, including OCT4 (B) and NANOG (C) expression, in hESCs. The hESCs were induced to differentiate into neural ectoderm by RA, SHH, Noggin and LIF, and the differentiating cells, after 9 days of treatment with extrinsic factors, changed their morphology and formed rosette type structures (D). Immunostaining for the neural markers Nestin (E), SOX1 (F) and Pax6 (G) and flowcytometry results for neural progenitor's marker genes (H) revealed the differentiation of hESCs toward neural cells. Further differentiation of rosette cells in the absence of Noggin, Shh, and RA for an additional week resulted in NT formation. NTs were then dissected manually (I) and, after dissociation, were replated onto laminin/poly-L-ornithine-coated dishes (J) for an additional two weeks in neurobasal medium supplemented with N2 and BDNF, GDNF, AA and cAMP to form a pool of matured neural cells (K). Immunostaining was performed and indicated the presence of differentiated neurons (L), astrocytes (M), serotonergic neurons (N) and dopaminergic neurons (O). Nucleuses are stained with DAPI and PI (propidium iodide). RA: Retinoic acid, Shh: Sonic hedgehog, LIF: Leukemia inhibitory factor, BDNF: brain derived neurotrophic factor, GDNF: Glial derived neurotrophic factor, AA: Ascorbic acid, NI: Neural induction, NE: Neural ectoderm, NT: Neural-like tube, DN: Differentiated neural cells.

**Figure 2:**
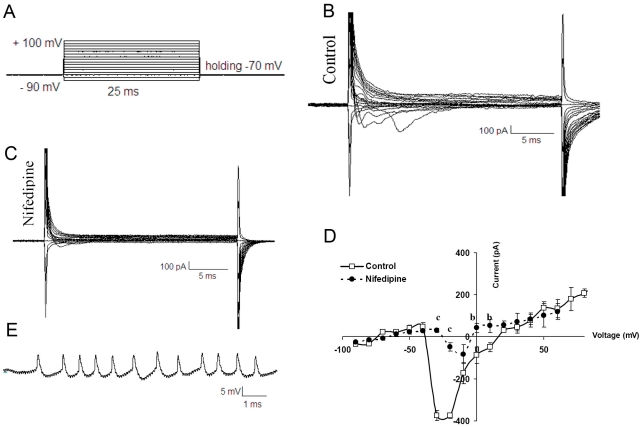
Inward currents recording in the differentiated neurons on two and four weeks after plating at DN stage. The electrophysiological protocol used for eliciting currents was as follows: voltage steps (25 ms duration) from a holding potential of -70 mV to a range of test potentials between -90 and +100 mV (10 mV increments) (A). Representative current tracing recorded from differentiated neurons before and after blocked by Nifidipine demonstrating the presence of Ca^++^ currents in these cells (B and C). I-V relationships for inward currents are shown (D). Action potential- like recordings in the differentiated neurons (E). b: P<0.01, c: P<0.001.

The morphology of feeder-free adhesive hESCs was compact cells with a high nucleus:cytoplasm ratio, resulting in cells with clear borders (Fig. 1A); they had a typical morphology and the key pluripotency markers OCT4 and NANOG (Fig. 1B, 1C). The hESCs were differentiated into NEs by Noggin and RA-mediated induction [8], which led to the appearance of rosettes with columnar cells (Fig. 1D) and NT formation. The differentiated cells, 9 days after induction (NE stage), expressed Nestin (Fig. 1E) (83.7±4.9%), Sox1 (Fig. 1F) (46.4±3.3) and Pax6 (Fig. 1G) (50.6±9.5%) (flowcytometry data Fig. 1H), which are neuroectodermal markers expressed during neural plate and NT formation [18]. We isolated hESC-generated NTs with a pipette (Fig. 1I). After dissociation, the cells were replated onto laminin/poly-L-ornithine-coated plates. Neural cell maturation was promoted by bFGF withdrawal and the addition of ascorbic acid, BDNF, GDNF, and cAMP (Fig. 1J). Within a few days, numerous processes grew, and neuronal migration began (Fig. 1K). The differentiated cells expressed neural markers (MAP2, GFAP, serotonin, and TH) that were detected by immunofluorescence staining (Fig. 1L-O). Quantification of the markers was performed as described previously [8]. To determine the functionality of the hESC-derived neurons we studied their electrophysiological properties (Fig. 2). In voltage clamp mode we evaluated voltage-activated ionic currents after plating. Voltage steps (25 ms duration) from a holding potential of −70 mV to a range of test potentials between −90 and +100 mV (10 mV increments) demonstrated inward currents (Fig. 2A). Inward currents that inactivated rapidly and were sensitive to Nifedipine, an L type Ca^++^ blocker (5 mM, student *t* test, P<0.001, n = 30) (Fig. 2B, 2C)

Current-voltage relationships showed that inward currents activated near −40 mV and peaked at −10 mV in matured cell two weeks after platting. (Fig. 2D) (n = 30).

The excitability of hESCs-derived neurons was assayed by whole cell patch clamping in the current clamp mode. Action potentials like responces were recorded after plating in 60% of matured cells (Fig. 2E).

### cDNA microarray analysis of hESC-derived neural cells

**Figure 3:**
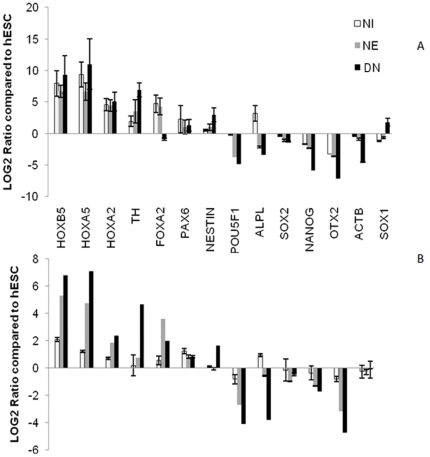
Confirmation of array data with quantitative real time RT-PCR for transcripts that were changed during differentiation. Real time PCR data (A) for stem cells specific transcripts (POU5F1, SOX2, ALPL and NANOG) that were down regulated during neural differentiation compared with array data (B). The expression of neural progenitor specific transcripts (FOXA2, Nestin and PAX6) and maturation markers (TH, HOXB5, HOXA5 and HOXA2) also compared. In all of the above transcripts, real time RT-PCR data confirmed the array results, just in the case of ACTB gene, that has a background expression in differentiated neurons in the array data, the real time data revealed that ACTB was down regulated in the neural differentiation. SOX1 expression also was not significantly changed in array (data was not shown) but has a background expression in differentiated neurons in the real time PCR data. Expression data were analyzed with the ΔΔCT method.

Three independent biological replicates of undifferentiated hESCs and cells from the NI, NE and DN stages were analyzed using whole genome microarray technology. As a first level of quality control, the transcriptional profiles of all replicate samples were assessed for biological reproducibility (Figure S1). All triplicate mRNA samples clustered together. Real time PCR was carried out to confirm the expression levels of genes selected from four different groups: (i) pluripotency markers, such as OCT4, SOX2, ALP, and NANOG (ii) genes that were upregulated during neural induction (e.g., Nestin, SOX1, PAX6), (iii) neural rosette cells (e.g., FOXA2), and (iv) genes that were upregulated in differentiated neurons (e.g., HOXA5, HOXB5, HOXA2, and TH). In 13 of 14 reactions, microarray-derived differential expression was confirmed at a confidence level of 92%. For one gene, ACTB, the background mRNA expression at maturation, detected by microarray, could not be confirmed by real time PCR (Fig. 3). For confirmation of the Nestin as a neural progenitor marker and Tyrosine hydroxylase (TH) as a transcript that was highly expressed in the differentiated neurons, protein expression level of Nestin and TH were analyzed by western blotting and results revealed that differences in mRNA levels actually reflect differences in protein expression in both cases (Figure S2).

### Distinct classes of transcripts are differentially expressed during neural differentiation

**Figure 4:**
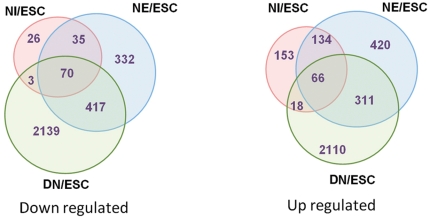
The diagram represents up- and down-regulated genes at different stages compared with hESCs. Significantly expressed transcripts (P<0.05, FC≥1.5) used as an input transcripts for clustering. The number of differentially expressed genes increased as differentiation progressed.

An analysis of transcriptome dynamics during differentiation revealed that 5955 transcripts were modulated during differentiation in at least one stage compared with hESCs (Table S2). As expected, the numbers of modulated genes increased during the differentiation of hESCs to MNs. While 505 and 1785 transcripts showed differential expression patterns in NIs and NEs, respectively, compared with hESCs, 5134 transcripts were modulated in MNs compared with hESCs (Fig. 4). While most (73%) of the modulated genes in NIs were up-regulated, only 48% (2,505) of regulated genes in the MNs were up-regulated. The minimum correlation of the expression patterns between stages was between the hESC and NI stages and between the NI and MN stages (rNI/MN = 0.68, rhESC/MN = 0.7) (Figure S1).

**Figure 5:**
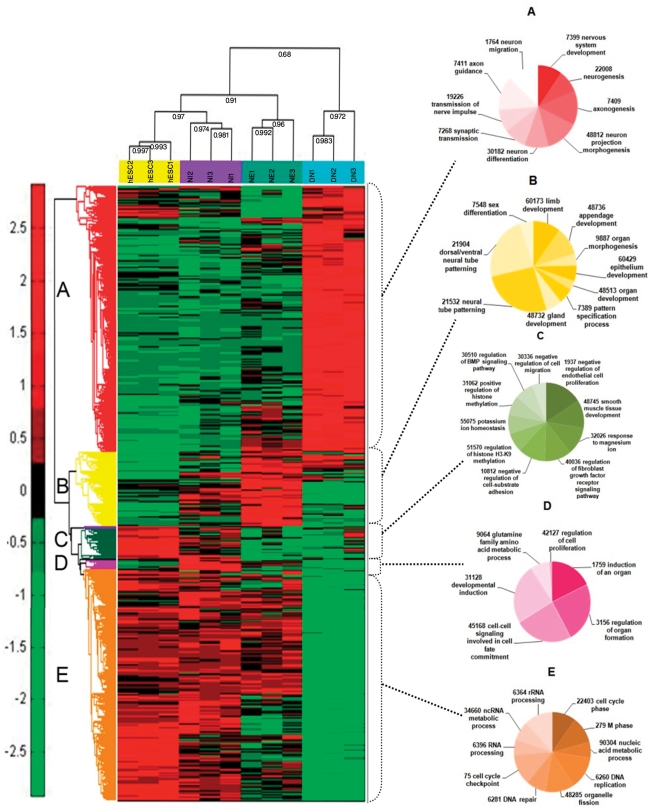
Hierarchical clustering of 5955 differentially expressed genes was performed using the mean signal intensity for each replicate. Three biological replicates of hESCs and differentiated cells at the NI, NE, and DN stages were compared and showed show high intraclass correlations compared with interclass correlations. Five distinct clusters were distinguishable based on the expression patterns of the different cell types. The differentially expressed transcripts were clustered into five expression groups, including (A) 2576 genes that were upregulated in DNs compared to other stages; (B) 720 genes that were upregulated in NEs; (C) 326 genes that were upregulated in hESCs and NIs compared with NEs and DNs; (D) 98 genes that were upregulated in Nis compared to the other stages; and (E) 2234 genes that were mainly down-regulated in differentiated neurons (Table S2).

The 5955 differentially expressed transcripts were categorized into five expression groups (Fig. 5), including (A) 2589 transcripts that were up-regulated in DNs compared to other stages, (B) 747 transcripts that were up-regulated in NEs, (C) 346 transcripts that were up-regulated in hESCs and NIs compared with NEs and DNs (D) 95 transcripts that were up-regulated in NIs compared to other stages, and (E) 2520 transcripts that were down-regulated in differentiated neurons (Table S2).

We performed a GO analysis of differentially expressed genes in different clusters using BINGO (v. 2.3) software; the hypergeometric test and the Benjamini and Hochberg false discovery rate (FDR) were used for statistical tests and multiple testing corrections, respectively. According to the expression patterns in the preliminary results, transcripts were categorized in two main clusters, first cluster contains transcripts that were up regulated in neural differentiation and second cluster contains undifferentiated associated transcripts, transcripts involved in neurogenesis, axonogenesis and gliogenesis were over represented in first class while cell cycle, DNA replication and repair, Mitosis and cell proliferation processes were over represented in undifferentiated cluster (Table S3).

Genes that were up-regulated in DNs (cluster A, Fig. 5, 6A) were mainly involved in neurogenesis, axogenesis, neuron migration, axon guidance, neurotransmitter secretion and synaptic transmission. These include the up-regulation of various genes related to differentiated neurons and glia, such as MAPT (microtubule associated protein tau) and other tubulin associated genes, synapse formation (SNAP25, SNAPB), septins, stathmins, intermediate filaments and axon guidance (ROBO-SLIT) molecules (Table S2). We also observed up-regulation of axon guidance cues and neurotrophins, including ROBO2 and ROBO3 and their receptors SLIT2 and SLIT3, Eph receptors (EFNB3, EPHA3, EPHA5, EPHA8, EPHB1, EPHB3) and their ligands, and semaphorin receptors (SEMA3E, SEMA4F, SEMA5B, SEMA6C) (Table S2).

**Figure 6:**
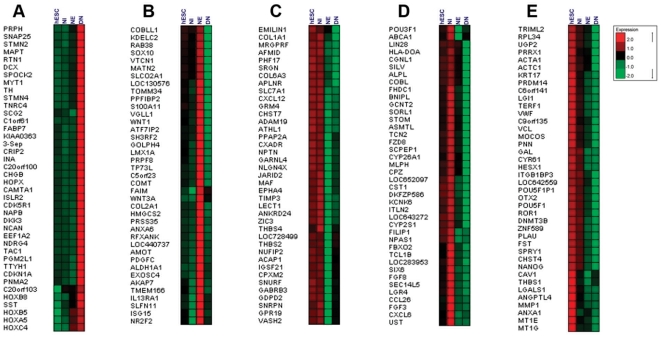
Expression patterns of representative genes from different expression clusters were presented in **Figure 3**. (A) transcripts that are highly up-regulated in DNs compared with the other stages; (B) transcripts that are enriched in NEs; (C) transcripts that are mainly up-regulated in hESCs and NIs; (D) genes that are enriched in NIs; and (E) highly abundant genes in hESCs.

The protachykinin gene (TAC1), tyrosine hydroxylase (TH) and the somatostatin gene (SST) were also among the highly expressed transcripts in differentiated neural cells. As the expression data show, peptide-releasing neurons (tachykinin, somatostatin, neurotensin peptides) coexist in culture with dopaminergic (DA) and gamma aminobutyric acid producing (GABAnergic)neurons, although their development may be different but this coexistence might be the result of neurotransmitter colocalization. There are many reports of colocalization of neurotransmitters in one neuron, especially neuropeptides, neurotensin and dopamine (DA) in dopaminergic neurons and also for somatostatin and GABA in GABAergic neurons [19], [20], [21].

The genes that were up-regulated in NEs (cluster B) were mainly involved in appendage development, epithelium development and pattern specification in the neural tube (Fig. 5, 6B). Differentially expressed genes in this group may be involved in basic molecular changes that underlie the conversion of progenitor cells to neural cells, including cell fate decisions and triggers for differentiation.

The expression of several progenitor marker genes, including LMX1a, MSX1, ALDH1A1 (dopaminergic progenitors), SOX10 (oligodendroglial precursors) and PCP4 (Purkinje neuron protein), as well as genes involved in tight junctions among epithelial cells (AMOT), and proliferation of neural progenitors, such as WNT1 and WNT3a, were higher in NEs. Transcripts of genes involved in neural tube development and patterning, such as ALDH1A2, FOXA2, VANGL2, ZNF358, are also among the genes that were up-regulated in NEs (Fig. 6B).

Several genes that were overrepresented in both hESCs and NIs were grouped together in cluster C (Fig. 5). These include genes involved in the negative regulation of cell migration and endothelial proliferation, the regulation of cell adhesion, histone methylation in the H3K9 position and the regulation of FGF and BMP signaling (Fig. 6C). Also represented in this group were genes for several adhesion molecules, including COL1A1 and COL6A3, neuroplastin, neuroligin and thrombospondins. These genes are involved in cell-ECM interactions, cell migration and axon guidance, which are crucial for hESC derived neural cells maintenance. The expression pattern of these genes revealed that the extracellular environment of hESCs might be more intimately related to the developmental lineage than to the biological properties of the neural plate. Neuroplastin (NPTN) is a glycoprotein that belongs to the immunoglobulin superfamily of cell adhesion molecules (CAMs). This gene is also involved in the long-term potentiation of hippocampal excitatory synapses through the activation of p38MAPK [22]. Recently, it was demonstrated that neuroplastin binds to and activates fibroblast growth factor receptor 1 (FGFR1) [23], and it may have a function in FGF signaling in hESCs. Neuroligin (NLGN4X) is a putative neuronal cell surface protein involved in cell-cell-interactions and may be involved in the formation and remodeling of central nervous system synapses [24]. It may also play a role in cell-cell interactions in hESC colonies. Thrombospondin family members (THBS4 and THBS2), which are down-regulated at the NE and DN stages, are adhesive glycoproteins that are involved in cell-cell and cell-ECM interactions [25]. THBS4 forms a pentamer and can bind to heparin and calcium, suggesting that this protein might be involved in local signaling in the developing and adult nervous system; its effect on the proliferation of endothelial cells is clear [25]. Another enriched protein in cluster C was JARD2, which modulates histone methyltransferase activity and promotes the recruitment of histone methyltransferase complexes [26] to their target genes. It also has a function in the neural tube fusion process [27]. The JARD2 protein binds DNA and mediates the recruitment of the PRC2 complex to target genes in ESCs. In ESCs, JARD2 associates with the PRC2 complex and inhibits trimethylation of Lys-27 of histone H3 (H3K27me3) by the PRC2 complex, playing a key role in the differentiation of ESCs and normal embryonic development [26].

Genes that are up-regulated in NIs (cluster D) were enriched in organogenesis, cell fate commitment, cell-cell signaling and developmental induction (Fig. 5, 6D). These include several well-known genes in the neurogenesis process; including Six6, FGF3, POU3F1, NPAS1, CYP26A1 and FGF8 (Fig. 6D). The higher expression levels of genes that modulate post mitotic neuron maintenance, such as FBXO2, may be required for the regionalization of neurons [28]. Several genes that were up regulated in NI are among a set of genes that are already known to be highly expressed in the developing CNS, including the Six6 gene, which is expressed abundantly in the brain, cerebellum and specific precursors of neural retina cells [29], and the FGF3 gene, which is expressed in the hindbrain and whose expression is required for hindbrain patterning [30]. POU3F1 also known as OCT6 is a member of the pou domain family of proteins and is involved in neural ectoderm formation; its expression is down regulated upon ESC differentiation and increases again during brain development [31].

The protein encoded by the NPAS1 gene is a member of the basic helix-loop-helix (bHLH)-PAS family of transcription factors and is specifically expressed in neural tissue [32]. NPAS1 in mice modulates the transcription of erythropoietin by binding to its enhancer region *in vivo*; thus, it indirectly controls oxygen-responsive elements during late embryogenesis and central nervous system development [33]. CYP26A1 plays a key role in retinoic acid (RA) metabolism. Many isoforms of this gene and other subunits of the P450 cytochrome are overrepresented at the NI stage, highlighting the importance of the retinoic acid (RA) metabolic pathway in the neural initiation stage [34]. Another gene, CPZ, modulates the WNT signaling pathway by cleaving some undefined protein or by binding to the WNT molecule [35]. FGF8 is also overexpressed in NI, and its expression is downregulated as differentiation progresses (Fig. 6). FGF8 is a paracrine factor that appears to have a function during dopaminergic neuron specification and proliferation [36]; it works cooperatively with SHH in the specification of midbrain neurons.

Neural rosette cells comprise neural progenitors from the neural crest and CNS neurons. Neural crest progenitors express S100A11, MSX1, TFAP2A, TFAP2B and ERBB3 abundantly in the rosette stage (Fig. 6B). During the early development of neural cells, the MEIS1 and MEIS2 homeobox genes can positively control PAX6 transcription and induce hESCs toward neuralization [37]. The extrinsic factor RA may trigger neural specific genes and induce the neural fate [38], and RA may also affect anterior-posterior pattern formation by inhibiting BMP signaling with Noggin, which induces the formation of neural ectoderm. Shh protein can induce Foxa2 and ventralize neural progenitors and, in a positive regulatory loop, FOXA2 can induce endogenous SHH and inhibit NKX2.2 [39] and also the serotonergic phenotype. Endogenous transcription of FGF8 resulting from RA exposure [40] can induce WNT1 expression that cooperatively with FGF8 can induce neural progenitors to differentiate into TH-producing cells.

A major group of differentially expressed genes includes transcripts that were down-regulated during differentiation (Fig. 5). This group includes genes involved in the cell cycle, mitosis and metabolism as well as genes involved in the development of other germ layer clusters (Fig. 6E). Several major ESC markers, including OCT4, NANOG, PRDM14, and GAL, were grouped in this cluster (Fig. 6E). Because OTX2, a gene involved in forebrain-hindbrain patterning, was also down-regulated during differentiation, the results suggest that posteriorized neurons were generated during differentiation in this study [41]. Matrix associated genes, including MMP1, THBS1, and ITGB1BP3, were also among the hESC enriched genes, suggesting that expression of these genes provides an environment conducive to the proliferation of stem cells. DNA methyltransferase genes and noncoding genes homologous to OCT4 control the epigenetic state of hESCs and are important classes of genes for stem cell maintenance. Some antagonists of FGF signaling, such as SPRY1, were also overrepresented in hESCs. SPRY1 is involved in cortical neuron pattern formation and inhibits caudal cell fates [42]; its role in hESCs with a high concentration of FGF is not clear and may be important for the fine tuning of FGF signaling in ESCs (Fig. 6E).

**Figure 7:**
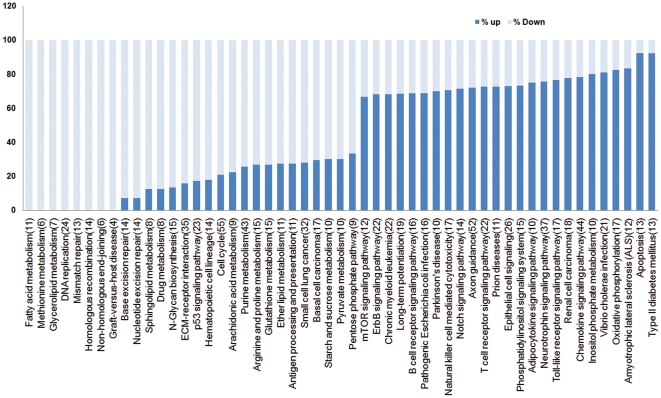
Pathway analysis of enriched transcripts in hESC (light blue) and differentiated cells (dark blue). Enriched transcripts searched against KEGG pathway database for finding the members involved in the signaling pathways, mostly transcribed members in each stage (up to 60%) in undifferentiated or neural differentiated cells compared and depicted in the figure. Common active pathways in both stages have not been shown.

Overall, our study found that the transcriptome is extremely complex and dynamic during early neural differentiation of hESCs. All transcripts that were modulated during differentiation were placed in upregulated and downregulated clusters and were analyzed using the KEGG pathway database and ClueGO software. With a two-sided hypergeometric test, genes were assigned to pathways. The pathway information for each cluster was analyzed, and pathways represented in each cluster were identified and compared. Our results showed that some transcripts that were involved in metabolic pathways were downregulated during differentiation. Pentose and glucuronate interconversions, fatty acid turnover (for membrane biogenesis), DNA replication, mismatch repair, recombination and immune response pathways were hyper expressed in hESCs, but not in neurons (Fig. 7, Table S4). Nucleotide metabolism and cell cycle pathway members were also highly expressed in hESCs. Genes involved in nitrogen metabolism, apoptosis, NOTCH signaling, axon guidance, neurotrophin signaling, Parkinson's disease and prion disease are highly abundant molecular pathways that were overrepresented in differentiated neural cells (Fig. 7, Table S4). In ESCs, mTOR signaling can stabilize OCT4, SOX2 and NANOG expression and can negatively control the induction of endoderm and mesoderm from ESCs. Inhibition of mTOR with rapamycin enhanced the expression of endoderm and mesoderm markers in treated EB and impaired the pluripotency of hESCs, but this effect was not observed in neural differentiation [43]. According to our results, mTOR signaling functions in neural induction. Transcripts associated with mTOR (S6K1) were upregulated during neurogenesis (Table S4). NOTCH signaling is also active in hESC-derived neural progenitors and has an important function in the proliferation and differentiation of NPCs. Inhibition of NOTCH can disrupt the maintenance of stem cell characteristics of NPCs [44]; [45], by suppressing the HES1 and HES5 genes, which negatively control the expression of the proneural genes MASH1 and NGN1 [46], [47], [48]. The NOTCH signaling members NOTCH1, DTX1, DTX3, DLL1, DLL3, HES5 and JAG2 were expressed more strongly at the NE stage compared to hESCs, and this stronger expression continued as the cells differentiated into final differentiated neurons. As shown previously, NOTCH signaling negatively controls neurogenesis in a stepwise process; in the first step, its activation leads to gliogenesis as opposed to neurogenesis, and in the second step, its activation promotes the production of astrocytes and inhibits the production of oligodendrocytes and neural fates [48].

Another pathway that confers one of the characteristics of NPs is the Toll like receptor (TLR) pathway. The TLR pathway is involved in the development of CNS and the innate immune system and functions in cell proliferation and NPC differentiation [49], [50]. The TLR pathway has an immunomodulatory effect on NPCs via the activation of TNF-alpha [51]. It seems that members of the TLR pathway (AKT1, CD14, FOS, IFNAR1, IL8, JUN, MAPK10, MAPK11, MAPK3, PIK3R1, PIK3R2, RELA, SPP1) are more highly expressed in NEs and DNs (Table S4). WNT signaling, MAPK signaling, Jak-STAT signaling, Hedgehog signaling, and TGF-beta signaling are active pathways in NEs and DNs, but these pathways do not share common proteins. The expression of WNT1 and most WNT receptors decreased, although the expression of WNT4, WNT7A, WNT7B and FZD9 increased with neural differentiation (Table S4). Neural progenitors (NPs) have some overlap with ESCs in the expression patterns of WNT pathway molecules. The DKK1 gene, an inhibitor of WNT signaling, was suppressed during neurogenesis. However, another inhibitor, DKK3, had an increased expression level, which may explain how different members of the WNT gene family may control differentiation of different cell types.

Metabolism associated genes are found in both ESCs and differentiated cells. Genes involved in the pentose phosphate pathway, galactose metabolism, ascorbate and aldarate metabolism and fatty acid elongation and metabolism have increased expression levels in stem cells. The beta-1,4-galactosyltransferase (B4GALT1) protein is an enzyme that participates in glycoconjugation, lactose biosynthesis, and galactose metabolism in liver cells. This enzyme is targeted to the plasma membrane of intestinal cells and absorbs galactose from the lumen; it may also function in adhesion [52]. Previous studies have shown that the B4GALT1 promoter has a binding site for E1AF, and induction of B4GALT1 expression by E1AF can lead to lung cancer [53]. Genes in the glycosphingolipid biosynthetic pathway are overrepresented in neural cells compared with ESCs. Glycosphingolipids (GSLs) are a group of bioactive glycolipids that includes cerebrosides, globosides, and gangliosides and are involved in cell adhesion, modulation of growth factor/hormone receptors, antigen recognition, and protein trafficking. GSLs are more active in neural tissues compared to hESCs, and their dysfunction may lead to the accumulation of GSLs and lysosomal storage diseases (LSDs) [54]. NOTCH signaling in neural system development has multiple functions. Not only can NOTCH switch a neural cell fate decision [55], but NOTCH signaling also plays an important role in the maintenance of neural stem cells [56]. NOTCH molecules are also needed for cell fate determination in hESCs as they differentiate into the three germ layers [57]. In this study expressed transcripts in this pathway were delta-like ligands (DLL1, DLL3), jagged (JAG2) and the receptor, NOTCH1. Activation of NOTCH1 can activate the transcription of HES5 and determine neural fate.

### Regulatory network of NPs at the NE stage

**Figure 8:**
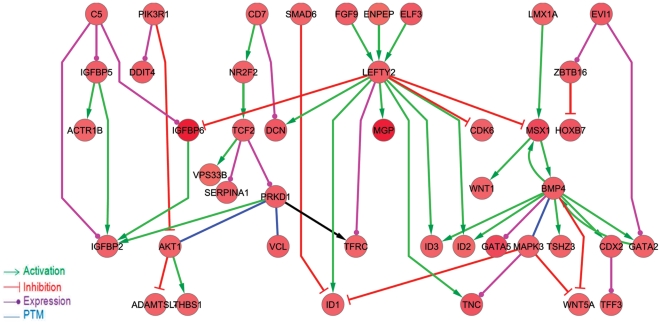
The gene regulatory network of 44 upregulated transcripts in neural rosette cells compared to hESCs. The STRING database was used to construct a regulatory network of differentially expressed transcripts. Then, a regulatory sub-graph was extracted from this network by selecting edges with inhibitory or activatory regulatory interactions. The visulation of networks was performed using Cytoscape. Green arrows indicate an activation mode, red arrows indicate an inhibitory mode, purple edges indicate an expression mode, and blue edges represent post-translational modification of source genes or target genes.

We established an interaction network of modulated genes at the NE stage using the STRING database. The full regulatory interaction network was composed of 96 nodes that were up- or down regulated more than 1.5-fold in rosette cells compared with hESCs. The data are presented in Figure S3. We then constructed a subnetwork of 44 nodes of NE-enriched genes (Fig. 8). Our results highlight the role of the TGFß family in the proliferation of neural progenitors. Genes in the TGFß superfamily are major players in the differentiation and maintenance of NPs. ID1 is a key gene in the proliferation of NPs [58] and is under the control of LEFTY2, another helix-loop-helix transcription regulator. The genes ID3 and ID2 are also activated by LEFTY2 and are important for the survival, proliferation [59] and specification [60] of neural crest progenitor cells. It seems that the LEFTY 2 gene is involved in NP proliferation (Fig. 8). MSX1 can activate BMP4, which works cooperatively with LEFTY2 to induce the transcription of ID2 and ID3. MSX1 is controlled by LMX1A and, in a positive feedback loop, can promote WNT1 expression. Reciprocally, WNT1 can positively activate LMX1A expression, leading to dopaminergic progenitor proliferation [61]. Temporal gene expression analysis of 55 cell cycle signaling molecules with significant modulation during differentiation revealed that CDK2, CDK4, CDK6 and molecules involved in the G2-S phase transition, including CDC2, CDC25C and MAD2L1, have higher expression levels in NEs compared to hESCs. TFDP1, another transcription factor that binds to EF1 and controls the transcription of EF1 target genes [62], is also upregulated in NEs (Figure S4).

In summary, transcriptional profiling during differentiation of hESCs to neural cells reveals systematic changes in the expression levels of transcription factors that control fate decisions, paracrine factors that coordinate the differentiation process, cell metabolism, cytoskeleton and genes in neurotransmitter secretion pathways. Our results clarify the gene expression changes that occur during differentiation of neural cells. Future studies will uncover functional changes in different neuronal subtypes and glia.

## Supporting Information

Figure S1 The expression levels of approximately 6000 transcripts are depicted in pairwise plots and correlations among samples, calculated separately for each sample pair (A: hESC, B: NI, C: NE, D: DN). The distance of points from the regression line indicates the diversity of expression between two samples. The X and Y axes represent the fold changes of transcript levels. The number of genes up or downregulated by more than 2-fold in compared samples is shown on the other side of the matrix.
(TIF)

Figure S2 Western blot analysis of 25 ug of total protein extracts from Royan H6 cells. Equal amounts of protein from total cell lysates of human ESCs and neural differentiated cells were subjected to SDS-PAGE followed by western blotting and visualization with an ECL detection kit. Bands were visualized with hyperfilm ECL (GE Healthcare, USA) and scanned with a GS800 densitometer (Bio-Rad, USA). ESCs, neural rosette and differentiated neural samples (three replicates) were analyzed with antibodies against Nestin and tyrosine hydroxylase (TH) proteins. In panel B, western blot and qRT-PCR results for Nestin and TH were quantified to compare the mRNA and protein levels at the NE and DN stages.
(TIF)

Figure S3 A regulatory network of 96 nodes, modulated by a 1.5-fold or greater up or downregulation in rosette cells compared to hESCs. The downregulated nodes are shown in green, and the upregulated nodes are shown with red circles. Green arrows indicate an activation mode, red arrows indicate an inhibitory mode, purple edges indicate an expression mode, and blue edges represent post-translational modification of source genes or target genes.
(TIF)

Figure S4 Cyclin dependent kinase and other transcripts associated with cell cycle progression and control, according to BINGO results, were clustered with Expander. The resulting image shows that the genes involved in cell cycle control are differentially expressed in embryonic stem cells and the neural progenitor state. Some of the transcripts are overexpressed in differentiated neurons and inhibit transition from the G1 phase (e.g., CDKN1B, CDKN2D).
(TIF)

Table S1 List of primers used for Real-Time PCR analysis.
(DOC)

Table S2 Normalized microarray data of 5955 transcripts modulated during differentiation in at least one stage compared with hESCs
(XLSX)

Table S3 BiNGO analysis of differentially expressed transcripts in neural differentiation. Over represented biological processes across transcripts which differentially expressed calculated compared to all annotation from human database
(XLSX)

Table S4 Pathway analysis of differentially expressed transcripts. Up-regulated transcripts in cluster 1 compared to down regulated transcripts in cluster 2, searched for KEGG pathways that they were involved.
(XLSX)
