# Correction: Comprehensive Gene Expression Analysis of Human Embryonic Stem Cells during Differentiation into Neural Cells

**DOI:** 10.1371/annotation/ba9082c9-69ee-43ac-81f0-5d71c3a01028

**Published:** 2011-08-05

**Authors:** Ali Fathi, Maryam Hatami, Vahid Hajihosseini, Faranak Fattahi, Sahar Kiani, Hossein Baharvand, Ghasem Hosseini Salekdeh

The correct definition of the acronym ACECR used in affiliations 1, 2, and 4 is "Academic Center for Education, Culture and Research." The corrected affiliations are:

1 Department of Molecular Systems Biology, Cell Science Research Centre, Royan Institute for Stem Cell Biology and Technology, Academic Center for Education, Culture and Research(ACECR), Tehran, Iran

2 Department of Stem Cells and Developmental Biology, Cell Science Research Centre, Royan Institute for Stem Cell Biology and Technology, Academic Center for Education, Culture and Research(ACECR), Tehran, Iran

4 Department of Developmental Biology, University of Science and Culture, Academic Center for Education, Culture and Research, Tehran, Iran

