# Supplementary figures and images for: Comprehensive Gene Expression Analysis of Human Embryonic Stem Cells during Differentiation into Neural Cells
Source: PLoS One. 2011 Jul 28;6(7):e22856. doi: 10.1371/journal.pone.0022856 (PMC3145766; doi:10.1371/journal.pone.0022856)

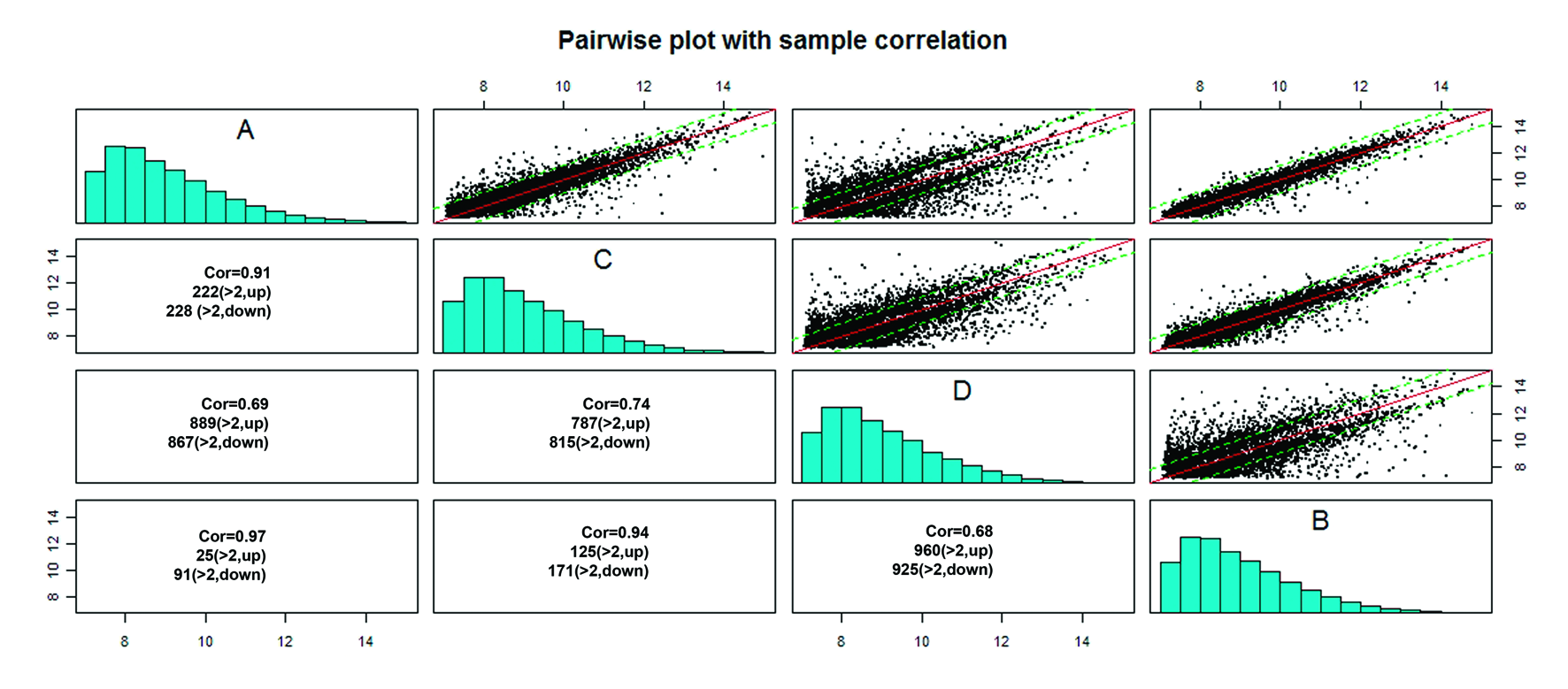

Supplement: Figure S1 — The expression levels of approximately 6000 transcripts are depicted in pairwise plots and correlations among samples, calculated separately for each sample pair (A: hESC, B: NI, C: NE, D: DN). The distance of points from the regression line indicates the diversity of expression between two samples. The X and Y axes represent the fold changes of transcript levels. The number of genes up or downregulated by more than 2-fold in compared samples is shown on the other side of the matrix. (TIF) [file pone.0022856.s001.tif]

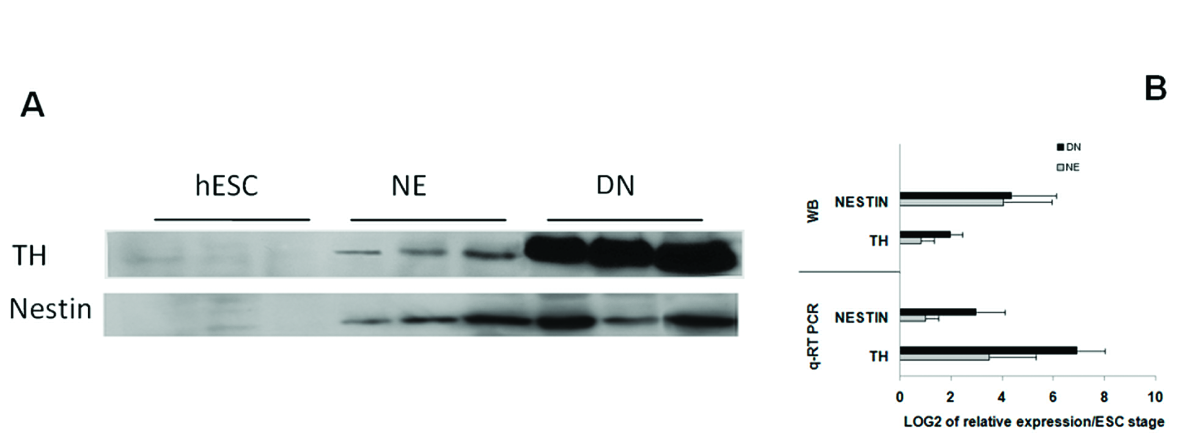

Supplement: Figure S2 — Western blot analysis of 25 ug of total protein extracts from Royan H6 cells. Equal amounts of protein from total cell lysates of human ESCs and neural differentiated cells were subjected to SDS-PAGE followed by western blotting and visualization with an ECL detection kit. Bands were visualized with hyperfilm ECL (GE Healthcare, USA) and scanned with a GS800 densitometer (Bio-Rad, USA). ESCs, neural rosette and differentiated neural samples (three replicates) were analyzed with antibodies against Nestin and tyrosine hydroxylase (TH) proteins. In panel B, western blot and qRT-PCR results for Nestin and TH were quantified to compare the mRNA and protein levels at the NE and DN stages. (TIF) [file pone.0022856.s002.tif]

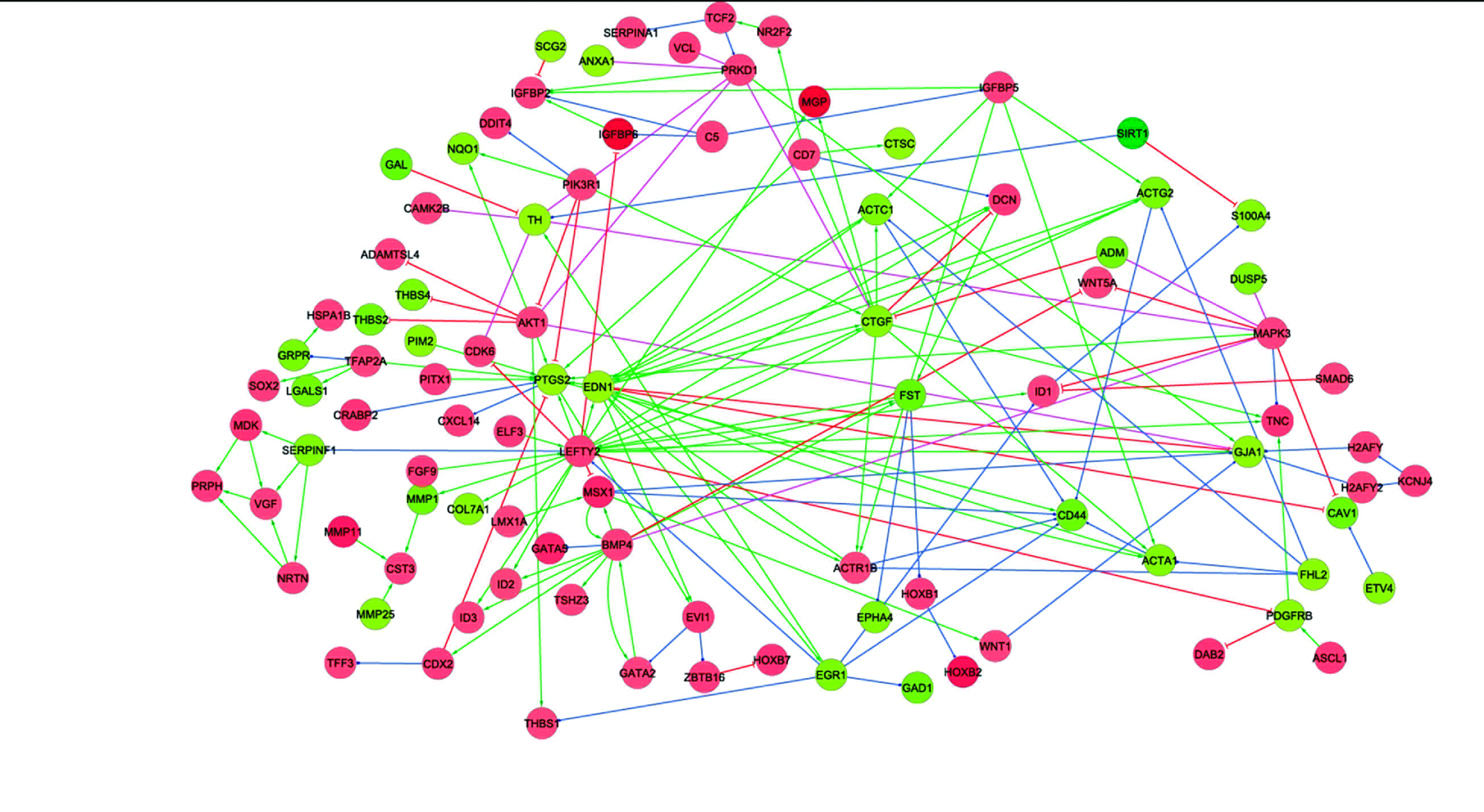

Supplement: Figure S3 — A regulatory network of 96 nodes, modulated by a 1.5-fold or greater up or downregulation in rosette cells compared to hESCs. The downregulated nodes are shown in green, and the upregulated nodes are shown with red circles. Green arrows indicate an activation mode, red arrows indicate an inhibitory mode, purple edges indicate an expression mode, and blue edges represent post-translational modification of source genes or target genes. (TIF) [file pone.0022856.s003.tif]

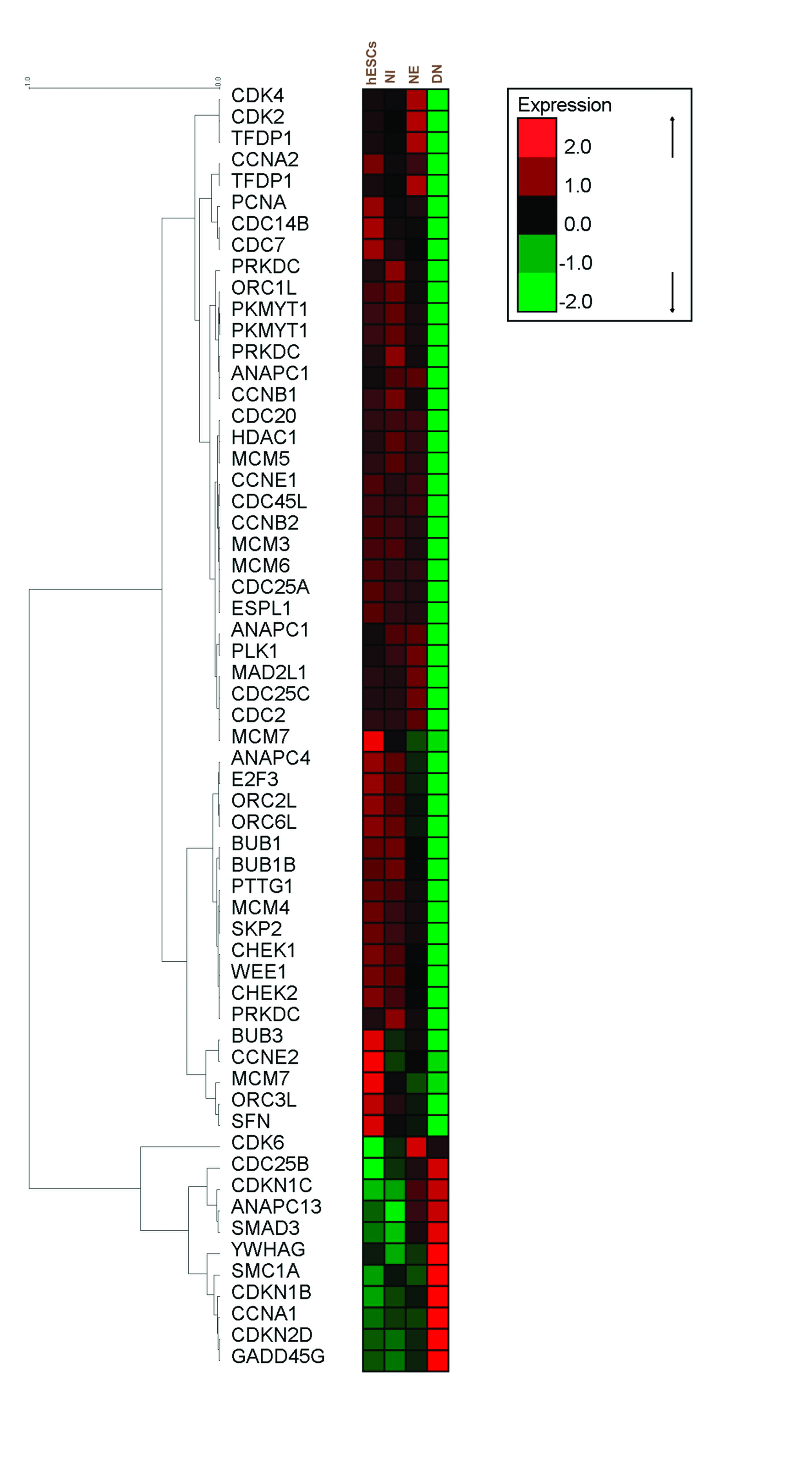

Supplement: Figure S4 — Cyclin dependent kinase and other transcripts associated with cell cycle progression and control, according to BINGO results, were clustered with Expander. The resulting image shows that the genes involved in cell cycle control are differentially expressed in embryonic stem cells and the neural progenitor state. Some of the transcripts are overexpressed in differentiated neurons and inhibit transition from the G1 phase (e.g., CDKN1B, CDKN2D). (TIF) [file pone.0022856.s004.tif]
