# Supplementary material for: Comprehensive Gene Expression Analysis of Human Embryonic Stem Cells during Differentiation into Neural Cells
Source: PLoS One. 2011 Jul 28;6(7):e22856. doi: 10.1371/journal.pone.0022856 (PMC3145766; doi:10.1371/journal.pone.0022856)
Supplement: Table S1 — List of primers used for Real-Time PCR analysis. (DOC) [file pone.0022856.s005.doc]

| **Gene** | **Forward Primer** | **Reverse Primer** |
| --- | --- | --- |
| ACTB | 5' AGC ACA GAG CCT CGC CTT 3' | 5' CAC GAT GGA GGG GAA GAC 3' |
| OCT4 | 5'CTGGGTTGATCCTCGGACCT3' | 5'CACAGAACTCATACGGCGGG3' |
| NANOG | 5'AAAGAATCTTCACCTATGCC3' | 5'GAAGGAAGAGGAGAGACAGT3' |
| ALPL | 5'GGCTGGAGATGGACAAGTTC3' | 5'CAGATTTCCCAGCGTCCTTG3' |
| SOX2 | 5'GGG AAA TGG GAG GGG TGC AAA AGA GG 3' | 5'TTG CGT GAG TGT GGA TGG GAT TGG TG 3' |
| SOX1 | 5' CACAACTCGGAGATCAGCAA 3' | 5' GGTACTTGTAATCCGGGTGC 3' |
| OTX2 | 5' CTC TGA ACC TGT CCA CCC 3' | 5' AGC AAG TCC ATA CCC GAA 3' |
| FOXA2 | 5' ATGCACTCGGCTTCCAGTAT 3' | 5' TGTTGCTCACGGAGGAGTAG 3' |
| PAX6 | 5' CGGTTTCCTCCTTCACAT 3' | 5' ATCATAACTCCGCCCATT 3' |
| HOXB5 | 5' TATACCCGCTACCAGACC 3' | 5' GTTGTCCTTCTTCCACTTCAT 3' |
| HOXA5 | 5' GAGCCACAAATCAAGCAC 3' | 5' CGCCGAGTCCCTGAAT 3' |
| HOXA2 | 5' AGGAGGACGAGGAAGAGA 3' | 5' ACTGGGAAACTTTGGGAG 3' |
| TH | 5' CAC CCA GTA TAT CCG CCA 3' | 5' CTT CTC AAT TTC CTC ATC CGA 3' |
| NESTIN | 5' CTCCAGAAACTCAAGCACC 3' | 5' TCCTGATTCTCCTCTTCCA 3' |

Supplementary table 1. List of primers used for Real-Time PCR analysis.
